# Application of Endoscopy in Improving Survival of Cirrhotic Patients with Acute Variceal Hemorrhage

**DOI:** 10.4061/2011/893973

**Published:** 2011-07-21

**Authors:** Yao-Chun Hsu, Chen-Shuan Chung, Hsiu-Po Wang

**Affiliations:** ^1^Division of Gastroenterology and Hepatology, Department of Internal Medicine, E-Da Hospital/I-Shou University, Kaohsiung 824, Taiwan; ^2^Division of Gastroenterology and Hepatology, Department of Internal Medicine, Far Eastern Memorial Hospital, New Taipei 220, Taiwan; ^3^Department of Internal Medicine, National Taiwan University College of Medicine, National Taiwan University Hospital, Taipei 100, Taiwan; ^4^Department of Internal Medicine, National Taiwan University Hospital, 7, Chung-Shan South Road, Taipei 100, Taiwan

## Abstract

Playing a central role in the modern multidisciplinary management of acute gastroesophageal variceal hemorrhage, endoscopy is essential to stratify patient at risk, control active hemorrhage, and prevent first as well as recurrent bleeding. Before endoscopic procedure, antibiotic prophylaxis along with vasoactive medication is now routine practice. Intravenous erythromycin effectively cleanses stomach and may improve the quality of endoscopy. The timing of endoscopy should be on an urgent basis as delay for more than 15 hours after presentation is associated with mortality. Active variceal bleeding on endoscopy in a patient with hepatic decompensation heralds poor prognosis and mandates consideration of aggressive strategy with early portosystemic shunting. Band ligation has become the preferred modality to control and prevent bleeding from esophageal varices, although occasionally sclerotherapy may still be used to achieve hemostasis. Addition of pharmacotherapy with nonselective beta blockade to endoscopic ligation has become the current standard of care in the setting of secondary prophylaxis but remains controversial with inconsistent data for the purpose of primary prophylaxis. Gastric varices extending from esophagus may be treated like esophageal varices, whereas variceal obliteration by tissue glue is the endoscopic therapy of choice to control and prevent bleeding from fundic and isolated gastric varices.

## 1. Introduction

Acute variceal hemorrhage (AVH) from esophageal varices (EV) or gastric varices (GV) is a devastating complication of portal hypertension. It is a leading cause of death in cirrhotic patients, particularly in those with hepatic decompensation. Early cohort studies observing the natural course of patients with AVH revealed that the short-term mortality rate was as high as 50%, with uncontrolled active hemorrhage and recurrent bleeding as the major causes of death [1–3]. As a witness of progress in modern medicine, the prognosis of AVH has remarkably improved for the last 3 decades, although the short-term mortality (conventionally defined as within 6 weeks of each episode) in recent series remained approximately 15–20% [4]. The improved outcome of cirrhotic patients with AVH probably results from advancement in the multidisciplinary approaches that include pharmacological therapy (vasoactive agents, antibiotic prophylaxis), endoscopic intervention (band ligation for EV, variceal obliteration for GV), transjugular intrahepatic portosystemic shunt (TIPS), and surgery. Being an essential part in the management of acute upper gastrointestinal (UGI) bleeding, endoscopy plays important roles in the confirmation of bleeders, stratification of risks, control of active hemorrhage, and prevention of the first and recurrent bleeding in cirrhotic patients with AVH [5]. The purpose of this paper is to provide a concise and updated review on the use of endoscopy in managing patients with AVH.

## 2. Preparation for Endoscopy in Cirrhotic Patients with Acute UGIB

Patients with AVH frequently present with unstable hemodynamics because bleeding characteristically occurs not only massively but also rapidly. Therefore, restoration of circulatory volume by intravenous fluid resuscitation should be carried out immediately at patients' arrival. Blood component therapy usually is needed to correct anemia and bleeding tendency (coagulopathy as well as thrombocytopenia). Vasopressor may occasionally be required to maintain hemodynamic stability. A quick assessment for the indications of airway protection by endotracheal intubation is mandatory, in that the concern of suffocating aspiration is substantial in patients with massive hematemesis, impaired consciousness, and delirious status. Ideally, risks of circulatory collapse and airway compromise should be minimized before patients are transported to endoscopy rooms. 

Intravenous administration of erythromycin prior to endoscopy may be considered in cirrhotic patients presenting with hematemesis, because brisk bleeding and large quantity of residual blood in the UGI tract often obscure endoscopic views, add difficulty of therapeutic intervention, and increase chance of aspiration. As a motilin receptor agonist, erythromycin induces peristalsis, stimulates gastrointestinal motility, and shortens gastric emptying time. The efficacy of erythromycin in cleansing stomach and thereby improving quality of endoscopy has been demonstrated in randomized controlled trials [6–8]. Recently, Altraif and colleagues reported in a double-blind randomized trial that erythromycin of 125 mg intravenously administered 30 minutes before endoscopy as compared with placebo significantly increased the proportion of a clear stomach (48.9% versus 23.3%, *P* < .01), decreased the mean procedural duration (19.0 minutes versus 26.0 minutes, *P* < .05), and shorten the hospitalized days (3.4 days versus 5.1 days, *P* < .02) in cirrhotic patients with AVH [7]. Besides, this medication appeared safe in these vulnerable patients without specific adverse reactions. Nevertheless, it remains unclear whether erythromycin also helps in controlling active hemorrhage, preventing recurrent bleeding, or adding survival benefit. So far, erythromycin infusion before endoscopy has not become a routine practice in most hospitals including ours. Finally, vasoactive agents (terlipressin, octreotide, and somatostatin) and prophylactic antibiotics before endoscopy unambiguously improve clinical outcomes and are now considered as an integral part of the evidence-based standard of care in cirrhotic patients presenting with acute UGI bleeding [9–11].

## 3. Timing of Endoscopy

**Figure 1:**
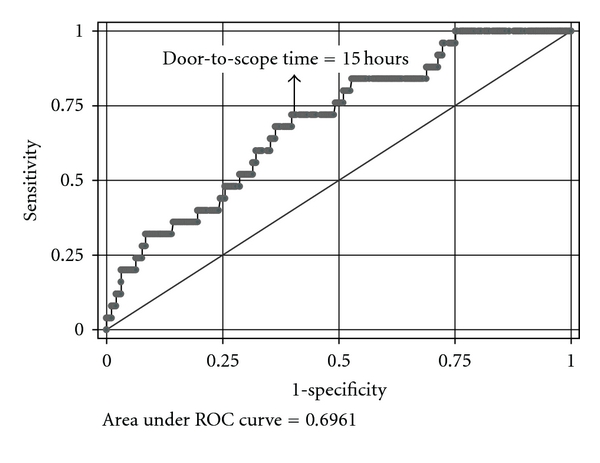
Receiver operating characteristic curve of “door-to-scope” time for in-hospital mortality. The area under curve is 0.696 (95% C.I. 0.595 ~ 0.797). The most optimal cut-off value (in integer) to predict in-hospital mortality was 15 hours, with sensitivity of 72.0% and specificity of 59.4% (adapted from [20]).

The optimal timing of endoscopy for patients with AVH has long been controversial. Earlier randomized controlled trials for patients with esophageal variceal bleeding found that endoscopic sclerotherapy as compared with vasoactive pharmacotherapy (terlipressin, somatostatin) was not more effective in terms of hemostasis rate, prevention of recurrent bleeding, or prolonging survival, but was associated with more adverse effects [12, 13]. D'Amico and colleagues thus concluded in a meta-analysis study that endoscopic therapy could be reserved for use after pharmacological treatment failed in EV bleeding [14]. However, this conclusion has become less clinically relevant after band ligation replaced sclerotherapy as the endoscopic therapy of choice for EV bleeding. Solid evidence supports the former was not only more efficacious but also safer than the latter [15]. Furthermore, since endoscopic plus pharmacological therapy is superior to either treatment alone and pharmacotherapy can be readily given before endoscopy [16, 17], it is no longer valid to suggest reserving endoscopic intervention (particularly band ligation for EV bleeding) after failure of vasoactive drugs. What remains unsettled is how urgently endoscopy should be performed in patients already receiving optimal medical therapy. Practice guidelines from the international conference (the Baveno workshop) for the management of AVH recommended UGI endoscopy be performed as soon as possible (<12 hours) after admission [18, 19]. However, this recommendation was supported not so much by objective data as by experts' rational consensus. To address this unresolved issue, we conducted a retrospective analysis of 311 consecutive cases with AVH to examine whether timing of endoscopy was associated with mortality [20]. We found that timing of endoscopy was correlated with in-hospital mortality (Figure 1). In multivariate analysis, delayed endoscopy (>15 hours after presentation to the hospital) was an independent risk factor associated with mortality (odds ratio 3.67; 95% confidence interval, 1.27~10.39). Our study, nonetheless, failed to demonstrate “the sooner the better” concept, in that the association between risk of death and endoscopy timing was nonlinear and mortality did not decrease with every hour earlier of endoscopy. Somewhat inconsistent with our observation, Cheung et al. from Canada reported that endoscopy timing was unrelated with clinical outcomes in hemodynamically stable AVH patients [21]. They found whether endoscopy was performed within 4, 8, or 12 hours within initial assessment at hospital did not influence recurrent bleeding, blood transfusion, need for rescue therapy, length of hospitalization, or mortality. Of note, only half of all patients with AVH were enrolled into analysis in their study, because the results of those with initial unstable hemodynamics were not reported [22]. In view of the understandable difficulty to perform a randomized trial to compare different endoscopy timings in this setting, the controversy will probably continue to exist. Based on currently available data, we believe the rule is to perform endoscopy within 15 hours of presentation, but meanwhile we also acknowledge there is no evidence to support rushing endoscopy in AVH patients, particularly in those with stable hemodynamics. Therefore, while delaying endoscopy for more than 15 hours should be avoided, endoscopists may wait in the first few hours to allow emergency resuscitation, optimal medication, and perhaps preparation for a cleaner stomach to be carried out.

## 4. Risk Stratification with and without Endoscopy

 Determination of bleeding source by upper GI endoscopy has important prognostic value in cirrhotic patients with acute UGI bleeding, since patients with variceal bleeding (definite or probable) fared significantly worse than those who bleed from other sources [23, 24]. In addition, active bleeding on endoscopy was shown to predict 5-day treatment failure and 6-week mortality [24, 25]. With regard to the risk prediction for patients with endoscopically confirmed AVH, measurement of hepatic venous pressure gradient (HVPG) is arguably the best method to stratify risk of untoward outcomes. It has been demonstrated that an initial HVPG > 20 mmHg most reliably identified those patients whose clinical course would evolve poorly [26]. Furthermore, a large body of evidence supports reduction of greater than 20% of the initial HVPG value convincingly indicates risk reduction in recurrent bleeding and mortality [27]. However, application of HVPG measurement is regrettably not widespread around the world, and in reality is not incorporated into daily practice in the vast majority of institutions. Fortunately, there is evidence suggesting that easily obtainable clinical variables, as compared with HVPG, may have similar accuracy in predicting failure of treatment during the acute phase of a bleeding episode (5 days) [28]. Among the various clinical parameters that have been investigated, indicators of hepatic reserve (Child-Turcotte-Pugh classification, model for end-stage liver disease (MELD) score), markers of bleeding severity (active bleeding on endoscopy, presentation with hematemesis, amount of blood transfusion, hemoglobin level), underlying liver disease or comorbidity (etiology, hepatocellular carcinoma, portal vein thrombosis), complications during bleeding episodes (encephalopathy, bacterial infection, renal dysfunction), and failure of initial treatment (uncontrolled active hemorrhage, recurrent bleeding) have been shown to predict clinical outcomes [20, 24, 29–32]. Endoscopic identification of AVH patients at risk of unfavorable outcomes may be crucial in guiding subsequent management. Garcia-Pagan and colleagues reported in a randomized trial that EV patients with hepatic decompensation (Child-Turcotte-Pugh scores between 7 and 13) and persistent bleeding at endoscopy would benefit from early TIPS performed within 72 hours [33]. The one-year survival rate was 86% versus 61% (*P* < .001) in patients randomized to early TIPS as compared with those who were assigned to receive optimal pharmacotherapy plus endoscopic band ligation. Therefore, it stands to reason that patients with actively bleeding and compromised liver function may require aggressive therapy to be implemented as early as a continuation to endoscopic therapy rather than as a rescue measure for treatment failure. 

 Although the prognostic factors for AVH have been extensively studied, those for cirrhotic patients with all sources of acute UGI bleeding remain sparsely explored. Undoubtedly, endoscopy is urgently indicated in cirrhotic patients presenting with UGI bleeding, but it takes time to resuscitate the patients, transfuse blood components, and administer intravenous medications. Therefore, risk stratification explicitly for variceal bleeding may not be applicable for clinicians managing patients in the emergency department, since not all cirrhotic patients bleed from varices. Previous studies that investigated prognostic indices independent of the source of bleeding not only incorporated endoscopic data but also allowed subjective criteria [23, 24]. In our opinion, criteria based on subjective judgment may not be reliable, particularly in the busy emergency setting. For example, uncovering and staging ascites and encephalopathy relies on expertise and is not free of interobserver variation [34]. We believe a useful stratification system in the setting of emergency room should ideally be built on simple, objective, and readily available parameters. To this end, we have retrospectively studied 542 consecutive episodes of acute UGI bleeding from 389 cirrhotic patients in order to develop a prognostic model consisting of pre-endoscopic clinical factors that were routinely available in the first hour at hospital [35]. We revealed that 6-week mortality was independently associated with male gender, hypoxemia on arrival, hepatocellular carcinoma and another malignancy, serum bilirubin, and prothrombin time (Table 1). The performance of a model built on these 6 variables was superior to the MELD score in predicting 6-week mortality, with *c* statistic of 0.84 and 0.71 respectively (*P* = .002). Presumably, earlier risk stratification may guide earlier modification of therapeutic approaches to improve the outcomes of those at risk. Further research is now warranted to elucidate how pre-endoscopic risk stratification will influence the early management for cirrhotic patients presenting with acute UGI bleeding.

## 5. Endoscopic Therapy for Primary Prophylaxis

 Screening endoscopy is mandatory to confirm the presence, to determine the size, and to uncover the stigmata of varices in cirrhotic patients, particularly in those with decompensated status [36–38]. Historically endoscopic sclerotherapy had been used in preventing the first bleeding from esophageal varices prior to the era of band ligation [39], but it is no longer recommended in this indication because the risk of complications may outweigh the potential benefits [40, 41]. EVL is technically infeasible for small esophageal varices defined as size <5 mm or F1 according to the classification proposed by Beppu et al. [38], whereas nonselective beta-blocker (NSBB) may slow the growth of small EV and thereby prevent the first variceal hemorrhage [42]. In patients with medium to large (or F2-F3) EV, risk of future bleeding is substantial and primary prophylaxis is indicated. Band ligation is as at least effective as NSBB for primary prophylaxis of EV bleeding [43–46]. The decision to use EVL or NSBB should be individualized according to the local resources and expertise, patients' preference and characteristics, tolerability of side effects, and contra-indications to either therapy. In fact, more than half of patients preferred EVL over NSBB use for fear of side effects from beta-blockade, such as light-headedness, shortness of breath, fatigue, and poor memory [47]. Because poor tolerability to NSBB is not uncommon and the response of HVPG to pharmacological therapy cannot be reliably assessed by clinical parameters, we usually perform EVL for primary prophylaxis in our institutes.

 There is no doubt that band ligation and NSBB are effective, respectively, to prevent first bleeding in the EV with medium to large size, but it remains unknown whether combination therapy with both treatment modalities is more effective than either therapy alone. Sarin et al. reported in a randomized controlled trial that propranolol plus EVL and EVL alone were not different in bleeding related death, although there was less recurrence of varices in the combination group (5.6% *versus *15.3%, *P* = .03) [48]. In a randomized trial conducted by Gheorghe et al., propranolol plus EVL as compared with propranolol alone resulted in lower rate of first bleeding from the high risk EV (6% *versus *31%, *P* = .03), and higher bleeding-free survival rate (96% *versus *69%, *P* = .04) during the 18-month followup in cirrhotic patients awaiting liver transplantation [49]. Nevertheless, Lo and colleagues demonstrated that EVL plus nadolol was not only not more effective that nadolol alone for primary prophylaxis of EV bleeding but also associated with more adverse events (68% *versus *40%, *P* = .06) [50]. As the controversy goes on, currently combination therapy with EVL plus NSBB cannot be recommended in patients whose EV has not bled.

## 6. Endoscopic Therapy to Control Active Bleeding

 Endoscopic therapy plays a pivotal role in the hemostasis of AVH. EVL is the recommended endoscopic therapy whenever feasible to control active EV bleeding, because it is unambiguously safer and more effective than sclerotherapy [51–53]. Occasionally, sclerotherapy may be substituted if EVL is technically difficult, for example, in a repeatedly ligated esophagus with scarred mucosa that is difficult to be sucked into the cap. It is important to carefully scrutinize the bleeding stigmata (e.g., hematocystic spot, white nipple) in those without ongoing bleeding at endoscopy. Localization of the origin of bleeding is essential for successful endoscopic therapy, inasmuch as EVL should be initiated at or just below the bleeding point. If the bleeder cannot be clearly localized, ligation may start at the gastroesophageal junction and then advance upward spirally. While active bleeding at endoscopy mandates immediate hemostasis, absence of ongoing hemorrhage during endoscopy should not be erroneously regarded as reassuring to reserve endoscopic therapy. In a randomized trial, Lo and colleagues compared EVL plus terlipressin versus terlipressin alone in cirrhotic patients presenting with acute inactive EV bleeding and demonstrated that EVL was effective in reducing 5-day rebleeding rate (0% versus 15%, *P* = .006), treatment failure rate (2% versus 24%, *P* = .002), and amount blood transfusion [54]. Therefore, EVL cannot be spared in cirrhotic patients with inactive bleeding EV at endoscopy if another bleeding source is unlikely.

 Injection therapy with tissue glue (e.g., N-butyl-2-cyanoacrylate and 2-octyl-cyanoacrylate) to obliterate varices has become the endoscopic treatment of choice for isolated gastric varices (IGV) and gastroesophageal varices extending beyond cardia (GOV2) [55]. Regrettably, there is considerably less data regarding the endoscopic therapy in controlling active GV hemorrhage, in contrast to the overwhelming evidence supporting the role of EVL in EV bleeding. Glue injection using cyanoacrylate for acute GV bleeding achieves high rates of immediate hemostatsis, eventual eradication, and low treatment failure-related mortality rate [56]. Consistent results from randomized trials provide convincing evidence to support the superiority of obliteration therapy over either sclerotherapy [57–59], or band ligation [60, 61]. While the techniques to achieve variceal obliteration vary in different institutes, it has been adopted in our daily practice to inject a mixture of N-butyl-2-cyanoacrylate and lipiodol (1:1) without contrast agent. Despite the efficacy and generally acceptable safety profile of injection therapy with tissue adhesives, thromboembolism infrequently occurs and represents the most fearful complication of cyanoacrylate injection that may potentially lead to infarction of multiple organs [62, 63]. Use of thrombin or fibrin has been explored in the management of acute GV bleeding with promising preliminary results [64–66]. Theoretical advantages of thrombin injection include biocompatibility and minimal mucosal damage, whereas possibility of transmissible infectious disease and excessive cost are major concerns. Before data from controlled trials comparing it with cyanoacrylate is available, thrombin injection should better be viewed as experimental and ideally be confined in the setting of clinical studies.

## 7. Endoscopic Therapy for Secondary Prophylaxis

As long as the portal hypertension persists, it is simply the natural course of varices to rebleed, with 1-year rebleeding rate approximating 60% [67]. Since gastroesophageal varices result from portal hypertension and occurrence of variceal hemorrhage depends directly on hydrostatic pressure of portal system (as reflected by HVPG), presumably the best treatment to prevent recurrent bleeding is to reduce the severity of portal hypertension, and that is the pathophysiological basis for the efficacy of NSBB. In view of the high recurrence rate, preventive measures for recurrent bleeding should be instituted right after acute bleeding episode is controlled. It is recommended that patients receive secondary prophylaxis before they are discharged from hospital for an bleeding episode, especially for those with large varices, red color signs, and decompensated cirrhosis [55]. 

Consistent with its superior role in primary prophylaxis and controlling active hemorrhage, EVL remains the preferred endoscopic treatment for secondary prevention of EV bleeding. EVL, again, outperforms sclerotherapy in this indication in terms of lower complication rate and higher efficacy [68–70]. Moreover, there is no evidence to embrace the addition of sclerotherapy to EVL. Singh et al. reported in a meta-analysis that combination of EVL and sclerotherapy as compared with EVL alone was not more effective in preventing recurrent EV bleeding, but was associated with higher complication events such as esophageal stricture [71]. In our opinion, endoscopic sclerotherapy has no role in the secondary prophylaxis of EV bleeding. With regard to variceal obliteration by tissue adhesives, there was a randomized trial demonstrating similar rebleeding rates between histoacryl injection and NSBB administration, but the former treatment was associated with a higher complication rate (47.6% *versus *10%, *P* < .03) [72]. Moreover, we are unaware of any trial comparing efficacy and safety of glue injection with that of EVL in the secondary prophylaxis of EV bleeding. In contrast to the scenario of primary prophylaxis, in which combination therapy with EVL and NSBB does not fare better than either therapy alone, combining endoscopic therapy plus pharmacological therapy is recommended in the setting of secondary prophylaxis. A meta-analysis including 23 studies showed that rates of rebleeding (both from all sources and specifically from varices) are lower with combination of endoscopic therapy (either sclerotherapy or EVL) plus drug therapy than with either therapy alone [73]. Therefore, cirrhotic patients recovering from acute EV bleeding should receive NSBB and have their varices eradicated by band ligation. In those who are unable or unwilling to undergo EVL, the addition of isosorbide mononitrites to NSBB appears a reasonable option.

Usually several sessions of banding ligation is needed in order to eradicate EV. However, the time interval of band ligation remains an unsettled issue. Although some studies proposed an interval of 1 to 2 weeks [69, 70, 74], others advocated an interval of 1-2 months of band ligation for obliteration of EV [75, 76]. Yoshida et al. found a short interval between sessions of EVL might even be detrimental by showing that the overall rates of variceal recurrence and additional treatment were both higher in patients with EVL at a biweekly interval than those with a bimonthly protocol [76]. Generally, we do not repeat sessions of EVL within 2 weeks because prior ligation-related mucosal ulceration may not have healed by that time and thereby may influence the following deployment of ligating bands. As far as efficacy is concerned, TIPS may be a more effective modality than endoscopic therapy to prevent recurrent bleeding. According to a meta-analysis, patients undergoing TIPS had a lower rebleeding rate than those receiving endoscopic treatment (19% *versus *47%, *P* < .001). The overall mortality, nevertheless, was not different [77]. The risk of hepatic encephalopathy, development of shunt stenosis, and the cost of a covered stent make TIPS traditionally considered as rescue therapy in patients with repeated AVH. However, as aforementioned in the section of risk prediction, early TIPS strategy (<72 hours) in high-risk patients improves survival significantly and may lead to paradigm shift in the future [33]. 

Despite the relative paucity of data in the efficacy and safety of using endoscopy to prevent recurrent hemorrhage from GV, tissue adhesives injection using N-butyl-cyanoacrylate is a reasonable choice for patients bleeding from IGV1 or GOV2, similar to control of acute bleeding, [55, 78]. For those who have bled from GOV1, either tissue adhesives injection or band ligation may be used, depending on the location of varices, technical feasibility, and expertise of the endoscopist. Unless it is technically infeasible, we recommend band ligation for EV and GOV1 at the same time.

## 8. Conclusion

 Endoscopy is essential in the modern multidisciplinary management of cirrhotic patients with AVH. Endoscopy should not be delayed for more than 15 hours as it is associated with increased risk of in-hospital mortality, although otherwise the data is insufficient for embracing “the sooner the better” belief, particularly in hemodynamically stable patients. Active bleeding at endoscopy in decompensated cirrhotic patients predicts poor outcomes and may warrant more aggressive treatment, such as early TIPS right after endoscopic therapy. Band ligation is the endoscopic modality of choice in primary prophylaxis, hemostasis of active bleeding, and secondary prophylaxis of EV bleeding. Although occasionally sclerotherapy may still be performed for hemostatic control of acute EV bleeding, it should no longer be used in the prophylactic setting. Tissue glue injection to attain variceal obliteration is now the preferred endoscopic therapy to control and prevent bleeding from fundic and isolated GV. The paucity of data in the management of GV warrants more research, particularly large controlled trials, to define the evidence-based standard of care. Even though substantial improvement has been achieved for the last several decades in the management of cirrhotic patients with AVH, there is undoubtedly plenty room for continuing improvement in this still highly lethal medical emergency.

## Figures and Tables

**Table 1:** Independent risk factors of 6-week mortality in cirrhotic patients with acute upper gastrointestinal hemorrhage, determined by multivariate logistic regression model.

	Adjusted odds ratio	95% confidence interval
Male sex	4.35	1.14 ~ 16.62
Hypoxemia^#^	9.42	3.65 ~ 24.30
HCC	2.31	1.12 ~ 4.78
Non-HCC malignancy	4.70	1.55 ~ 14.26
Bilirubin (per mg/dL)	1.07	1.02 ~ 1.13
INR (per unit)	2.88	1.28 ~ 6.51

^#^Hypoxemia is defined as peripheral oxygen saturation less than 95%; HCC: hepatocellular carcinoma; INR: international normalized ration (adapted from [35]).
